# Intraoperative detection of blood vessels with an imaging needle during neurosurgery in humans

**DOI:** 10.1126/sciadv.aav4992

**Published:** 2018-12-19

**Authors:** Hari Ramakonar, Bryden C. Quirk, Rodney W. Kirk, Jiawen Li, Angela Jacques, Christopher R. P. Lind, Robert A. McLaughlin

**Affiliations:** 1 Sir Charles Gairdner Hospital, Nedlands, Western Australia, Australia.; 2 School of Surgery, University of Western Australia, Crawley, Western Australia, Australia.; 3 ARC Centre of Excellence for Nanoscale Biophotonics, Faculty of Health and Medical Sciences, University of Adelaide, Adelaide, South Australia, Australia.; 4 Adelaide Medical School, Faculty of Health and Medical Sciences, University of Adelaide, Adelaide, South Australia, Australia.; 5 Institute for Photonics and Advanced Sensing, University of Adelaide, Adelaide, South Australia, Australia.; 6 Institute for Health Research, University of Notre Dame, Fremantle, Western Australia, Australia.

## Abstract

Intracranial hemorrhage can be a devastating complication associated with needle biopsies of the brain. Hemorrhage can occur to vessels located adjacent to the biopsy needle as tissue is aspirated into the needle and removed. No intraoperative technology exists to reliably identify blood vessels that are at risk of damage. To address this problem, we developed an “imaging needle” that can visualize nearby blood vessels in real time. The imaging needle contains a miniaturized optical coherence tomography probe that allows differentiation of blood flow and tissue. In 11 patients, we were able to intraoperatively detect blood vessels (diameter, >500 μm) with a sensitivity of 91.2% and a specificity of 97.7%. This is the first reported use of an optical coherence tomography needle probe in human brain in vivo. These results suggest that imaging needles may serve as a valuable tool in a range of neurosurgical needle interventions.

## Teaser

A “smart” brain biopsy needle containing a tiny imaging probe is able to warn neurosurgeons about nearby blood vessels.

## INTRODUCTION

Stereotactic brain biopsies are a minimally invasive procedure used to obtain samples of intracranial tissue for diagnostic purposes, most commonly related to brain tumors. Approximately 80,000 new cases of primary brain tumor are diagnosed, and 14,000 brain biopsies are performed each year in the United States (*1*, *2*). Hemorrhage is the most frequent and devastating complication associated with this procedure. Perioperative hemorrhage is associated with rates of transient and permanent morbidity of 1.7 to 8.5% and 1.4 to 4.8%, respectively, and mortality rates of 0.6 to 2.8% (*3*–*7*).

The standard clinical practice is to identify blood vessels at risk of injury on preoperative imaging, using either contrast-enhanced magnetic resonance imaging (MRI) or x-ray computed tomography. Frameless stereotactic navigation techniques, guided by preoperative imaging, are then used to direct the biopsy needle trajectory to sample the target lesion, while avoiding vasculature or eloquent brain tissue (*8*).

However, these standard techniques do not eliminate the risk of hemorrhage, as they are limited by their spatial resolution in the detection of smaller vessels and do not account for errors in stereotactic precision. Precision in stereotactic navigation is dependent on the quality of the preoperative imaging, accuracy of the image-to-patient registration, mechanical error of the trajectory guide, and accuracy of intraoperative probe tracking (*9*, *10*). Preoperative imaging can be impeded by patient motion, limited spatial resolution, and a range of imaging artifacts. Image-to-patient registration can be affected by geometrical lesional changes, such as rapidly evolving neoplasms, cerebrospinal fluid loss, cerebral edema, or cystic decompression (*9*, *10*). These factors can result in brain shift. While less notable during burr hole–based procedures than in open surgery, Ivan *et al*. (*11*) quantified the shift as up to 10.1 mm. In addition, accuracy of intraoperative probe tracking can suffer stereotactic error or line-of-sight deficiencies (*12*–*14*). Either one or a combination of these factors can contribute to increased hemorrhage risk.

Several techniques have been developed to perform neurosurgical procedures with improved guidance. Intraoperative MRI allows for real-time updates during surgery; however, this technique is often impractical due to workflow interruptions and can be very expensive (*15*–*17*). Intraoperative ultrasound has been used widely throughout neurosurgery; however, it is impractical with regard to its use in stereotactic brain biopsies due to its large probe size and poor spatial resolution for detection of vessels smaller than 2 mm in diameter (*18*–*20*). Indocyanine green is used in neurosurgery for blood vessel visualization but requires a direct line of sight to the vessel (*21*). Optical techniques, including laser speckle imaging (*22*–*24*), laser Doppler (*25*), and diffuse correlation spectroscopy (*26*), have been used to visualize superficial blood flow in an open surgery setting. A forward-facing laser Doppler flowmetry probe has been integrated into a stereotactic system for guidance of deep brain stimulation electrodes and demonstrated in human surgery (*27*–*29*). This system has been adapted for use during brain biopsies, although it has not been integrated with a brain biopsy needle (*30*). Instead, the optical probe was inserted along the planned needle trajectory and then fully retracted, and the biopsy needle was subsequently inserted. Preliminary work has also explored the integration of a multifiber optical spectroscopy system into a brain biopsy needle, but this work has not progressed to assessment in humans (*31*).

Here, we explore the use of optical coherence tomography (OCT) to intraoperatively detect vessels at risk of intracranial hemorrhage. OCT is an optical imaging technique commonly used in ophthalmology (*32*) and cardiology (*33*, *34*). OCT is label free, requiring no exogenous contrast agent, and instead is dependent on the endogenous optical scatterers in the tissue. Tissue is illuminated using near-infrared light, and the backscattered optical signal is reconstructed into a structural image of the tissue, typically at a spatial resolution of 5 to 20 μm, although resolutions as small as 1 to 2 μm have been achieved (*35*, *36*). OCT provides depth-resolved imaging, where the backscattered signal from different depths is extracted using low-coherence interferometry (*37*) by optically interfering the backscattered light beam with a reference light beam reflected along a path of known optical path length. Using terminology adapted from the field of ultrasound imaging, an A-scan (amplitude scan) refers to a single OCT acquisition and provides a one-dimensional (1D) measurement of optical backscatter along the direction of the light beam. A sequence of A-scans may be acquired to form a 2D image of the tissue, referred to as a B-scan.

A limitation of OCT is its image penetration depth. This is typically restricted to 1 to 1.5 mm in turbid tissue by the confounding effects of optical scattering and absorption (*38*–*40*). However, brain biopsy needles are inserted to a depth of several centimeters, well beyond the range of OCT. This has necessitated the development of miniaturized fiber-optic probes, which may be incorporated into the hypodermic needle to provide guidance near the distal tip of the needle as it is inserted at depth (*41*). Previous studies have demonstrated the use of these imaging needles in a range of tissues, including breast (*42*–*44*), lung (*45*, *46*), and brain (*47*, *48*). While the design of these probes is well established, their use in human studies is very limited, with most work restricted to animal or ex vivo human tissue, and no previous studies have demonstrated their use for in vivo human brain.

We have developed a miniaturized fiber-optic probe using OCT for the detection of cerebral blood vessels. This probe was integrated into a standard commercial neurosurgical stereotactic biopsy needle and tested intraoperatively in 11 live human patients undergoing craniotomy. The probe detected surface blood vessels (diameter, >500 μm) with a sensitivity of 91.2% and a specificity of 97.7% and was also able to successfully detect deep blood vessels. To the best of our knowledge, this is the first successful in vivo human trial to examine detection of blood vessels in the brain using OCT.

## RESULTS

### A brain biopsy imaging needle

**Fig. 1:**
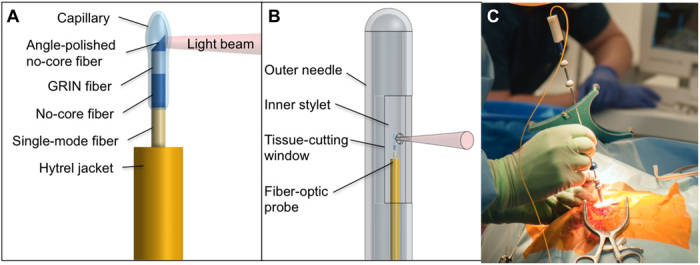
Imaging needle design. (**A**) Schematic of the distal end of the fiber-optic probe. (**B**) Schematic of the distal end of the imaging needle, showing the outer needle, inner stylet, and fiber-optic probe. (**C**) Photo showing the imaging needle inserted into a human brain during surgery (photo credit: The Audio Visual Production Unit, Sir Charles Gairdner Hospital).

We developed an optically guided biopsy needle capable of OCT imaging (Fig. 1), integrated into a standard clinical brain biopsy needle (Passive Biopsy Needle Kit, Medtronic), with the goal of identifying the presence of blood vessels before biopsy tissue cutting. As the needle was inserted, the system displayed the OCT image (providing a structural view of the tissue microstructure) overlaid with the local measurements of flow, providing near real-time feedback of vessel detection to the surgeon. Blood vessels were automatically detected through the use of an algorithm to analyze the rate of speckle decorrelation in the OCT image. Details of the needle design and blood vessel detection algorithm are given in Materials and Methods.

We devised two clinical protocols with their respective aims:

(1) Superficial vessel protocol: to evaluate the ability of the probe to detect cerebral vasculature against a gold standard.

(2) Deep vessel protocol: to demonstrate that the probe was able to detect cerebral blood vessels at depth and illustrate its integration into clinical workflow.

### Superficial vessel protocol

To quantify the ability of the probe to detect cerebral vasculature, it was important to devise experiments where a gold standard is available, including vessels not reliably visible on standard preoperative imaging. To achieve this, we compared the OCT measurements of superficial blood vessels on the brain surface (after craniotomy) against a gold standard of intraoperative photographs acquired during OCT imaging. This involved 11 patients undergoing craniotomy for indications including tumor, dermoid, and arteriovenous malformation excision. Patient demographics were seven (63.3%) females and four (36.4%) males. Mean (SD) age was 62.2 (15.3) years. We acquired 160 data points across 11 patients (44 controls, 55 arteries, and 61 veins).

For each patient, craniotomy and durotomy were performed, and the imaging needle, fitted within the outer cannula of a standard brain biopsy needle, was placed over a range of surface blood vessels within the operative field. Two OCT measurements were acquired for each blood vessel, and a photograph was concurrently acquired with a sterile scale bar in the field of view while the imaging needle was positioned on the vessel. This enabled easy identification of the measured blood vessel during analysis. During surgery, each vessel was analyzed by an experienced neurosurgeon (H.R. or C.R.P.L.) and classified as either artery or vein, based on macroscopic morphological features, including vessel wall appearance, pulsatility, compressibility, and color, and by tracing it back to its parent vessel. The classification was subsequently confirmed in postoperative analysis of the photograph. Postoperatively, the blood vessel diameter was measured from the photograph using imaging software [ImageJ (*49*)]. Control measurements with no visible vasculature were obtained from dura, arachnoid, and cerebral cortical surfaces.

We performed differentiation of blood vessel from nonvascular control tissue using a fully automated analysis algorithm that compared the average OCT speckle decorrelation across the two acquisitions against a threshold value. We used the same threshold across all datasets. Scans where the average speckle decorrelation was above the threshold were categorized as containing vessels. This was then tabulated against the photographic gold standard to assess accuracy of the algorithm. There was a significant difference between the median of the average speckle decorrelation values computed across vessel scans and across control scans (*P* < 0.001).

We scanned a wide range of vessel diameter sizes from 110 μm to 1.2 mm, with a median size of 500 μm. We performed analysis on two subsets of the data: larger vessels and all vessels. There is no accepted threshold vessel diameter that defines a clinically significant vessel, and other factors such as location and territorial supply contribute to clinical importance. We arbitrarily chose a cutoff, such that vessels with a diameter greater than the median of 500 μm would be denoted as the larger vessels, allowing us to exclude extremely small vessels that are less likely to be clinically relevant. This resulted in 101 data points across the 11 patients (44 controls, 14 arteries, and 43 veins with a diameter of >500 μm). Sensitivity, specificity, positive predictive value, and negative predictive value for both analyses (larger vessels and all vessels) are summarized in Table 1.

**Table 1:** Results of the superficial vessel protocol.

	**Sensitivity**	**Specificity**	**Positive predictive value**	**Negative predictive value**
Vessels > 500 μm (57 vessels and 44 controls)	91.2%	97.7%	98.1%	89.6%
All vessels (116 vessels and 44 controls)	86.2%	86.4%	94.3%	70.4%

**Fig. 2:**
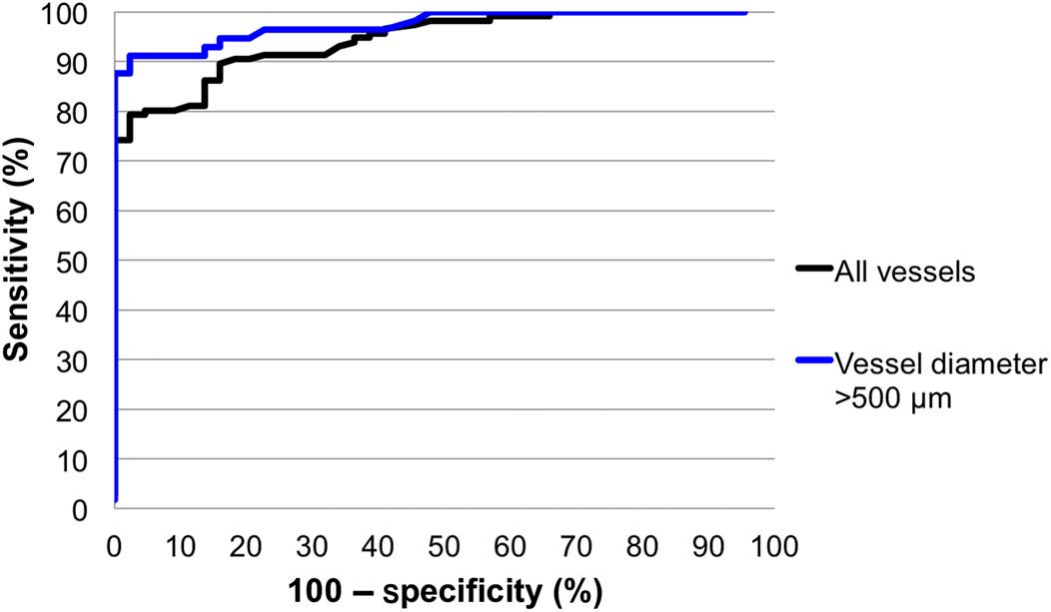
ROC curves for vessel detection. Blue indicates analysis of larger vessels (diameter, >500 μm). Black denotes analysis of all vessels (diameter, 110 μm to 1.2 mm).

By varying the threshold value of average speckle decorrelation that is used to differentiate vessels from control tissue, optimal sensitivity and specificity for blood vessels with a diameter of greater than 500 μm were found to be 91.2 and 97.7%, respectively. A receiver operating characteristic (ROC) curve for the threshold values was calculated (Fig. 2), giving an area under the curve (AUC) [95% confidence interval (CI)] of 0.975 with *P* < 0.0001. No significant difference was observed in the detection frequency of arteries and veins (*P* = 0.319).

When including all vessels (down to the minimum diameter of 110 μm), we chose a lower threshold value of average speckle decorrelation to achieve an optimal sensitivity and specificity of 86.2 and 86.4%, respectively. When analyzing all vessels, the ROC had an AUC (95% CI) of 0.945 with *P* < 0.0001. We partially attribute this decrease in sensitivity and specificity to the reduced speckle decorrelation present in extremely small vessels. However, we also note that accurately rolling the imaging needle across extremely small vessels in an in vivo setting can be challenging, and some vessels may not have been detected because of experimental error in positioning of the needle. As with the larger vessels, there was no significant difference between detection frequency of arteries and veins (*P* = 0.591).

**Fig. 3:**
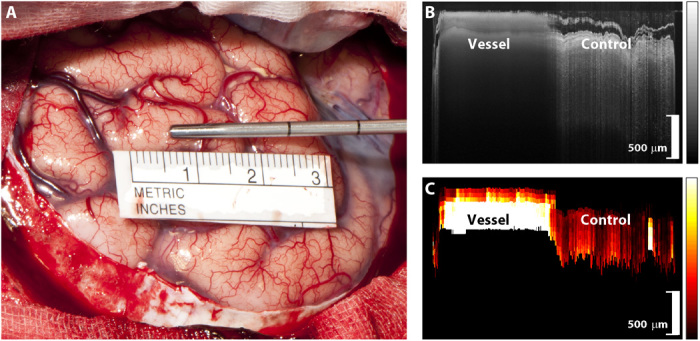
Superficial vessel protocol. (**A**) Photo of an imaging needle being rolled over a vessel (vessel diameter, 650 μm). The imaging window (not visible) is 0.5 mm from the end of the needle and is facing toward the tissue. A sterile scale bar is present and allows subsequent estimation of vessel diameter. (**B**) OCT B-scan consisting of A-scans acquired as the needle is manually rolled across the tissue. Tissue surface corresponds to the top of the OCT image, with depth increasing as we move down the image. (**C**) Speckle decorrelation image calculated from the OCT scan. Areas of high decorrelation are shown as white, and areas of low decorrelation are shown as dark red. These values are averaged and compared against a threshold to detect the presence of a vessel in the scan. Vertical scale bars, 500 μm (photo credit: The Audio Visual Production Unit, Sir Charles Gairdner Hospital).

**Fig. 4:**
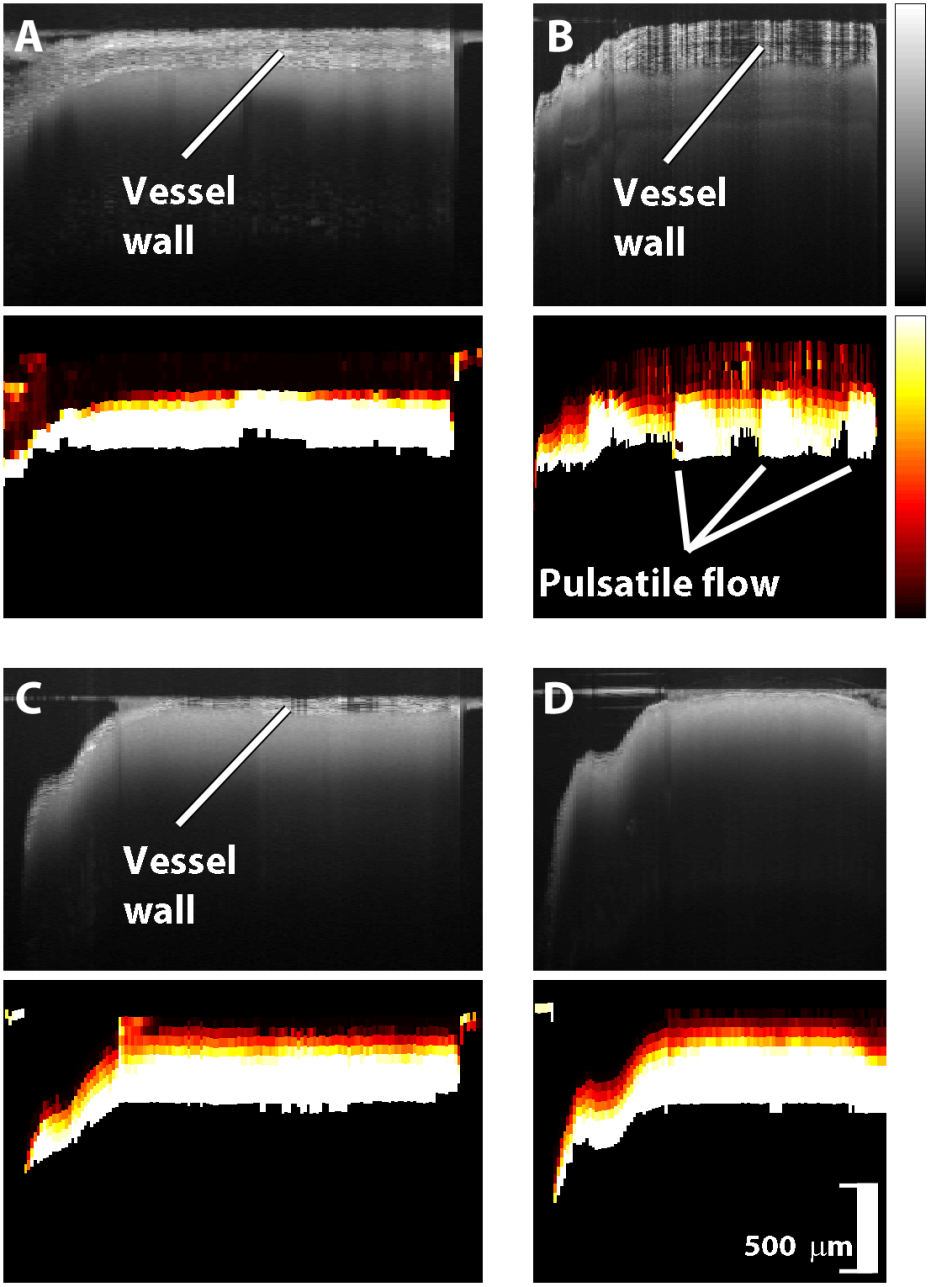
Blood vessel scans. In each pair of images, the OCT scan is shown on top and the corresponding speckle decorrelation image is shown below. (**A** and **B**) Arteries (vessel diameter, 720 and 260 μm). (**C** and **D**) Veins (vessel diameter, 1000 and 800 μm). Vertical scale bar, 500 μm.

**Fig. 5:**
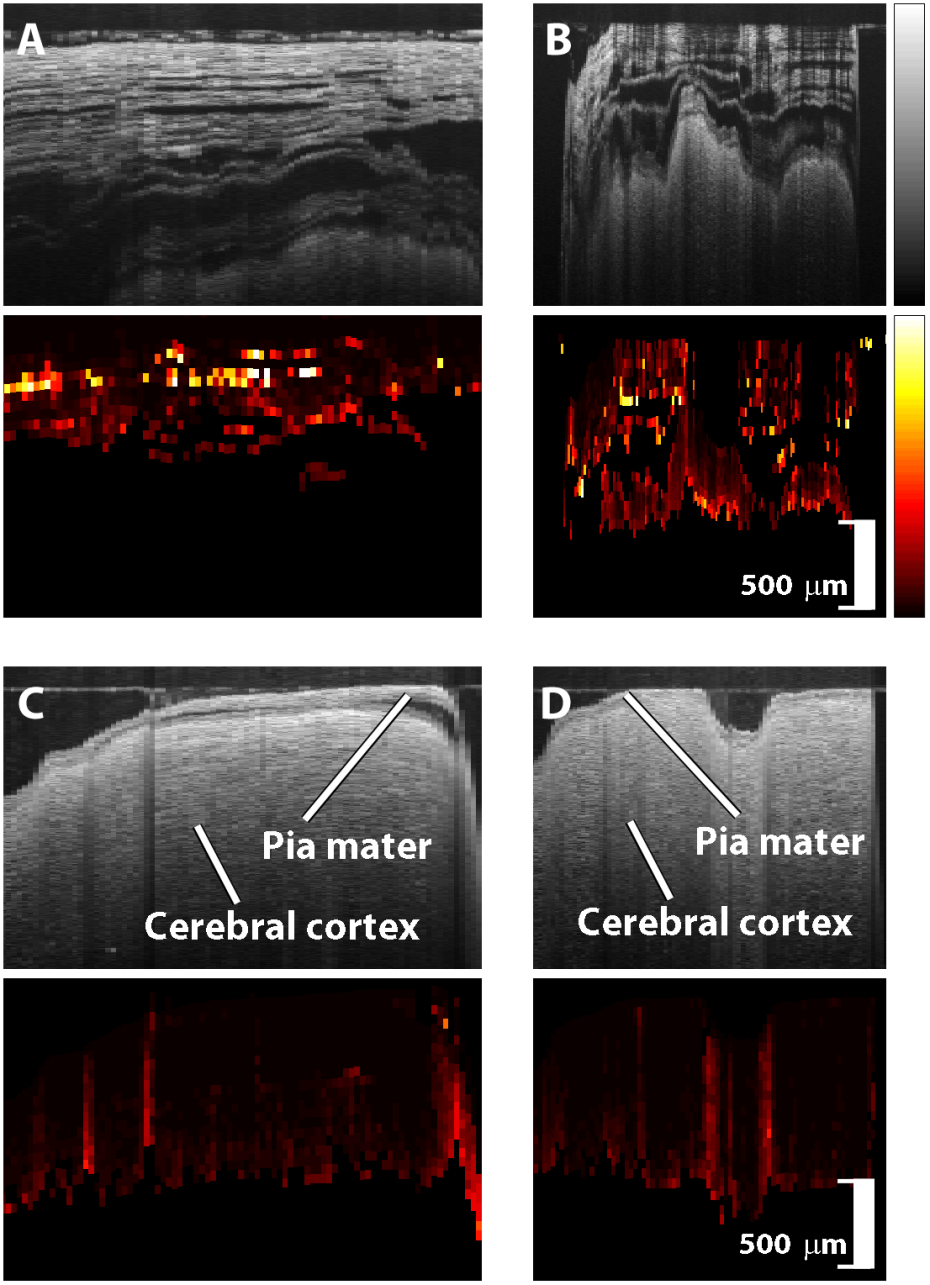
Control scans. In each pair of images, the OCT scan is shown on top and the corresponding speckle decorrelation image is shown below. (**A** and **B**) Arachnoid tissue. (**C** and **D**) Cortical tissue. Vertical scale bars, 500 μm.

**Fig. 6:**
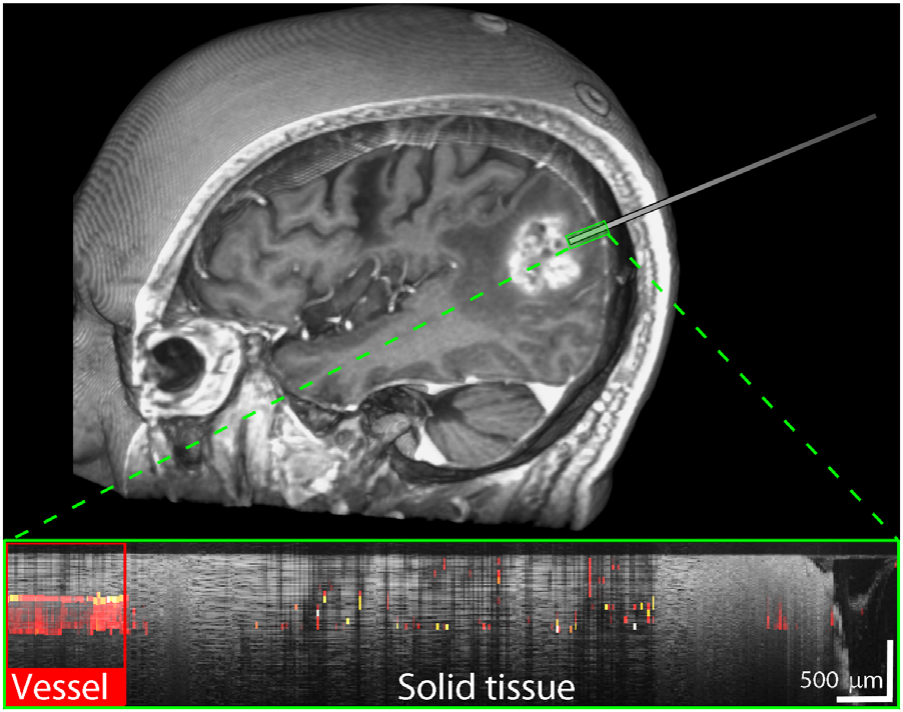
Deep vessel protocol. 3D volume rendered MRI scan showing needle trajectory in a 69-year-old male patient. The small green rectangle indicates the region in which OCT scanning was performed. The inset shows the overlaid OCT (gray) and speckle decorrelation (color) images, with the automatically delineated vessel indicated by a red rectangle.

**Fig. 7:**
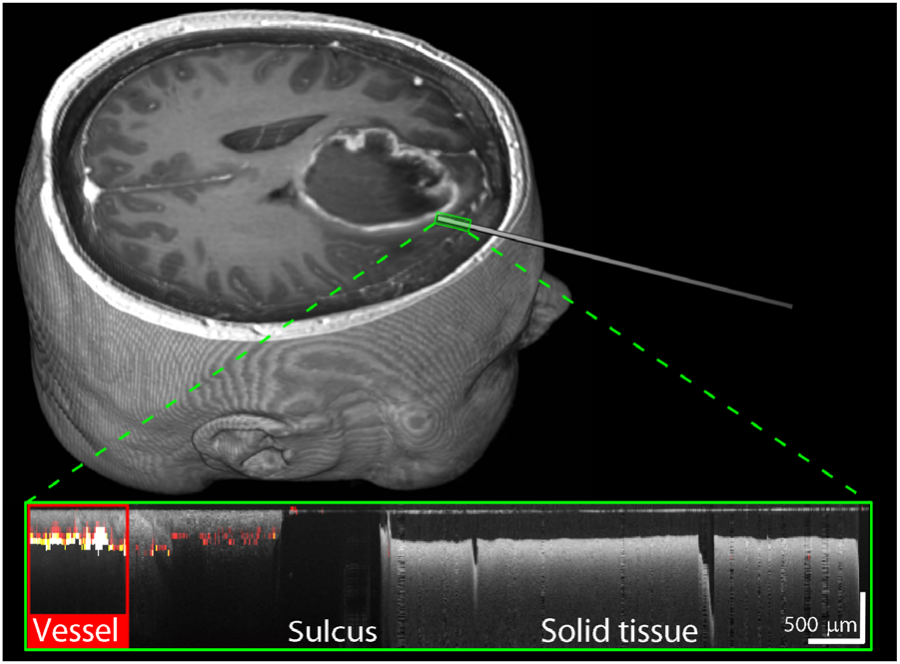
Deep vessel protocol. 3D volume rendered MRI scan showing needle trajectory in a 58-year-old female patient. The small green rectangle indicates the region in which OCT scanning was performed. The inset shows the overlaid OCT (gray) and speckle decorrelation (color) images, with the automatically delineated vessel indicated by a red rectangle.

**Fig. 8:**
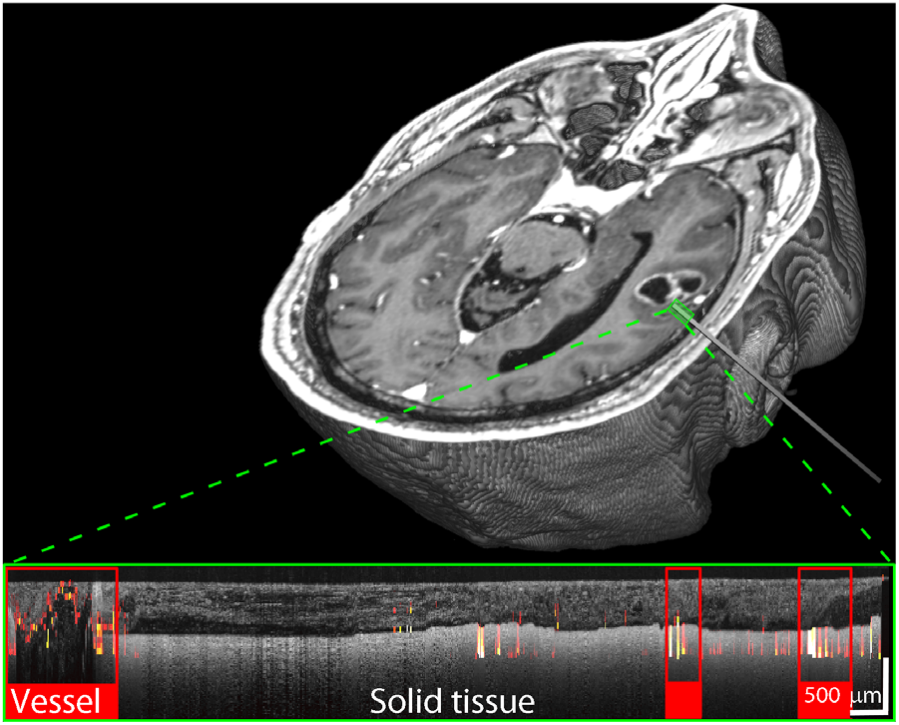
Deep vessel protocol. 3D volume rendered MRI scan showing needle trajectory in a 64-year-old male patient. The small green rectangle indicates the region in which OCT scanning was performed. The inset shows the overlaid OCT (gray) and speckle decorrelation (color) images, with multiple automatically delineated vessels indicated by red rectangles.

Figure 3A shows the exposed cortical surface of a patient undergoing tumor resection following craniotomy and durotomy. Surface blood vessels are evident on the cortical surface as the imaging needle is being rolled across a vessel to acquire a measurement. The corresponding OCT structural (Fig. 3B) and speckle decorrelation images (Fig. 3C) are shown. The left half of these scans was acquired over the vessel, and the right half was acquired over adjacent nonvascular tissue. Note the difference in the texture of the OCT, originating from rapid decorrelation of the intensity values where there is blood flow. The measured speckle decorrelation is color coded in Fig. 3C, with areas of high speckle decorrelation color coded in white and yellow, graduating to dark red for low speckle decorrelation. The speckle decorrelation color bars in Figs. 3 to 8 are all plotted with the same scale. However, the units are arbitrary units rather than absolute flow speed, as it was not possible to sufficiently correct for variations in the speed with which the neurosurgeon manually moved the needle. The size of the scale bars in Figs. 3 to 8 is 500 μm, assuming a refractive index of 1.36 for brain tissue (*50*).

Figure 4 shows representative OCT scans of vessels and the corresponding speckle decorrelation images. Note the thick vessel wall visible in the arteries shown in Fig. 4 (A and B). As expected, the walls of the veins shown in Fig. 4 (C and D) are much thinner. A pulsatile variation is visible in the speckle decorrelation of the artery of Fig. 4B, corresponding closely to the patient’s heart rate of 61 bpm as measured intraoperatively with electrocardiography (Datex-Ohmeda, GE Healthcare). The pulse rate was estimated from the OCT by manually measuring the average number of A-scans per pulse, scaled by the A-scan acquisition rate of 23 kHz. Pulsatile flow was observed in some arteries but was not present in any veins.

Representative examples of control scans are presented in Fig. 5, showing both arachnoid tissue (Fig. 5, A and B) and cortical tissue (Fig. 5, C and D). The pia mater could be delineated in some cortical scans. Small, focal areas of high speckle decorrelation are visible in the control scans, typically corresponding to the edges of structures or noise in the measurements. However, the average speckle decorrelation value across the entire scan was significantly lower than that for scans of vessels (*P* < 0.001).

### Deep vessel protocol

For the final three patients enrolled in the study, we demonstrated deep blood vessel detection with the imaging needle. Our aim was to ascertain whether the probe could detect vessels at depth, similar to its proposed clinical application.

All three patients had grade IV astrocytomas. One deep vessel per patient was selected on preoperative MRI immediately adjacent to the tumor. The needle trajectory chosen was the most direct to the target vessel, while remaining within the region scheduled for subsequent resection after imaging. A single burr hole was made, and the imaging needle, encased within the outer cannula of a standard brain biopsy needle, was targeted to the vessel using stereotactic navigation (StealthStation, Medtronic). Once at target depth, the needle was rotated to ascertain the presence of the target vessel, and the OCT speckle decorrelation pattern was observed intraoperatively. The needle was then withdrawn, and the standard procedure for craniotomy and resection of tumor proceeded as planned. The presence of the vessel was detected with an automated image processing algorithm described in Materials and Methods. For all three patients, the imaging needle was able to detect blood vessels at depth.

Figure 6 demonstrates the case of a 69-year-old male undergoing craniotomy and resection of a left parietal high-grade glioma. The 3D volumetric rendering of the MRI illustrates the needle trajectory, with a small green rectangle delineating the 8-mm trajectory from the surface of the cortex to the vessel, over which the OCT scan was acquired. The total insertion distance from the outer surface of the skull was 21.9 mm. The inset shows the OCT scan overlaid with the speckle decorrelation image. A vessel, previously identified on the MRI scan (diameter on MRI, 1.7 mm), is identified at the deepest part of the insertion as an area of high speckle decorrelation, overlaid in red.

Figure 7 demonstrates a second deep tissue insertion for a 58-year-old female undergoing right frontal craniotomy. The inset shows overlaid OCT and speckle decorrelation scans over a trajectory distance of 7.5 mm, terminating at the vessel. The total insertion distance was 25.3 mm. The targeted vessel (diameter on MRI, 1.7 mm) appears as a region of high speckle decorrelation. The OCT shows that the needle trajectory passed through a fluid-filled void, indicating a sulcus and appearing as an area of low backscatter in the OCT. We infer that the void is likely to be fluid filled because an air-filled void would show high optical backscatter (large OCT signal) at the interface where there is a large refractive index change between air and tissue, but no such interface is evident. The needle trajectory subsequently passed through a superficial region of cortical tissue (labeled solid tissue) with high backscatter.

Our final deep insertion was acquired on a 64-year-old male undergoing left temporal craniotomy and resection for glioblastoma multiforme. A vessel adjacent to the tumor was targeted (diameter on MRI, 1.6 mm). The OCT scan was acquired over the deepest 11.5 mm of the insertion, extending from the surface of the cortex. The total insertion distance was 27.2 mm. The vessel is shown detected in the OCT scan at the deepest part of the scan (red rectangle on the far left in the OCT inset of Fig. 8). Note that two smaller, more superficial vessels were also identified in the OCT data and are both delineated in Fig. 8. These vessels are below the 1-mm resolution of the MRI scan and were not visualized in preoperative imaging. More detailed MRI images of the targets for these three individuals are given in figs. S1 to S3.

## DISCUSSION

We have demonstrated that OCT speckle decorrelation is capable of accurately identifying cerebral blood vessels in live human patients. Furthermore, we have shown that this can be done with minimal disruption to current clinical workflow. Only minor changes in surgical technique are required, as the OCT probe is readily interchangeable with the inner stylet of a standard brain biopsy needle.

In clinical practice, the imaging needle has the potential to minimize hemorrhage by avoiding blood vessel sampling through the tissue-cutting window. By minimizing hemorrhage, the complication profile of the procedure is minimized. Furthermore, diagnostic yield could be further improved by facilitating safer acquisition of a greater number of tissue samples without increasing hemorrhagic risk (*51*). This, in turn, would contribute to improving cost-effectiveness by reducing the number of repeat procedures that are required.

Adverse clinical outcomes associated with stereotactic biopsies and hemorrhagic complications are primarily related to the vascular territory supplied by that blood vessel, as well as any potential mass effect that intracranial hemorrhage may cause (*52*, *53*). There are no published data reflecting what vessel diameter may be clinically relevant with regard to hemorrhagic complications. As the brain is highly vascular, it is almost inevitable that very small vessels may be damaged during the insertion of a stereotactic needle. We were able to reliably visualize blood vessels greater than 500 μm in diameter. We note that some speckle decorrelation algorithms provide an estimate of blood flow speed (*54*) and, combined with an estimate of vessel diameter, this could provide an indication of blood flow volume that may lead to more reliable indication of clinical significance.

Optical imaging techniques are well suited for use in an intraoperative setting due to the lack of ionizing radiation, the real-time nature of the images, and the ability to miniaturize the imaging hardware. Previous work has explored the use of laser Doppler imaging (*25*) and variants of laser speckle imaging (*22*–*24*, *55*) to monitor cerebral blood flow without the need for an exogenous contrast agent. Alternative approaches have involved video imaging of a fluorescent contrast agent, indocyanine green, to provide an indication of perfusion (*21*, *56*). However, all of these optical approaches have been restricted to assessing superficial flow in open surgery because of the lack of penetration of the light in turbid tissue. Optical techniques working in the diffuse regime, both with (*57*) and without (*26*) contrast agent, achieve a greater penetration depth but with a very poor spatial resolution that is unable to identify individual vessels at risk during an intervention. Our approach of miniaturizing the imaging probe and encasing it within the needle offers an alternative solution to the issue of limited penetration depth in high-resolution optical techniques, placing the imaging probe at the point of tissue sampling. A similar approach has been demonstrated using a miniaturized, forward-facing laser Doppler flowmetry probe, both in deep brain stimulation (*27*–*29*) and brain biopsy interventions (*30*). However, our design is the first to combine the optical probe into the biopsy needle, integrating it into the clinical workflow and avoiding the need for additional needle insertions.

The device presented here performs imaging through the tissue-cutting window. However, intracranial hemorrhage may also occur if the needle penetrates a vessel during insertion. While there are no statistics comparing the incidence of hemorrhage from tissue cutting versus needle insertion, we note that hemorrhage rates in interventions where no tissue cutting is performed are substantially reduced, suggesting that many bleeds occur during tissue cutting. For example, in deep brain stimulation, rates of symptomatic hemorrhage vary from 0.5 to 2.1% (*58*, *59*), compared with 1.7 to 8.5% for biopsy (*3*–*7*). There is also the potential to incorporate a forward-facing OCT probe within the biopsy needle to reduce risk during needle insertion. These designs have already been demonstrated for non-neurosurgical applications (*44*), and our team has demonstrated a forward-facing optical design similar to that used in this current paper (*60*). We note that the diameter of the distal focusing optics of the probe is only 250 μm, and the body of the probe is 900 μm in diameter due to a protective Hytrel jacket around the fiber. This small size allows for both a forward-facing probe and a side-facing probe to be incorporated into the stylet of a standard brain biopsy needle, to provide imaging to mitigate hemorrhage arising from both needle insertion and tissue cutting.

These small imaging probes have the potential to be integrated into other areas of probe-based neurosurgery, such as electrode placement in deep brain stimulation, where hemorrhage may occur by direct trauma during needle insertion. This could potentially increase utilization of this procedure across additional indications by improving the risk profile. Previous studies have also demonstrated the use of OCT in deep brain animal models for precise anatomical localization, with the potential to aid electrode placement (*48*). In addition to tissue biopsy applications, these miniaturized imaging probes may enable better targeting of therapeutic injections, and earlier work by our group has demonstrated a 22-gauge needle (outer diameter, 720 μm), integrating a fiber-optic OCT probe with a second channel for injection of a fluid (*61*).

Other groups have developed small form factor probes for neurosurgery, integrating different optical modalities (*30*, *62*–*66*), including Raman spectroscopy and 5-aminolevulinic acid/protoporphyrin IX (5-ALA/PpIX) imaging, to differentiate healthy from pathological tissue. It would be feasible to integrate our blood vessel detection capabilities with these other modalities, and a combined OCT and fluorescence probe has already been proposed by our group (*67*). This would allow the imaging needle to have multimodal capabilities, with the potential to optimize biopsy sampling by using fluorescence or the Raman spectrum to identify when malignant tissue is adjacent to the tissue-cutting window, while using OCT to identify blood vessels at risk of hemorrhage.

OCT speckle decorrelation detection of cerebral blood vessels holds great promise to reduce hemorrhagic risk from stereotactic brain biopsy. Limitations of the technology do, however, exist, predominantly the small penetration depth of approximately 1 to 1.5 mm in optically scattering tissue (*38*–*40*). This depth is not limited by the power of the imaging light but, instead, by the confounding effects of multiple scattering. However, even with this limited imaging depth, the device still has the potential to reduce the risk of hemorrhage. In conclusion, this work presents a technique for real-time blood vessel detection integrating with current clinical workflow with >90% sensitivity and specificity and sets the stage for further clinical trials to evaluate its safety and efficacy.

## MATERIALS AND METHODS

### Study design

The objective of this study was to assess the capability of OCT to detect intracranial blood vessels in humans. We hypothesized that imaging needles can differentiate vascular from nonvascular tissue in a neurosurgical setting. A customized fiber-optic probe integrated into a commercial stereotactic biopsy needle was used in adult neurosurgical patients (*n* = 11) at Sir Charles Gairdner Hospital, Western Australia. The indications for craniotomy included tumor, arteriovenous malformation, and intracranial dermoid resection. Exclusion criteria included people under the age of 18, people unable to give informed consent, non–English-speaking people, and patients with a bleeding disorder. Informed consent was obtained from each patient according to the Sir Charles Gairdner Group Human Research Ethics Committee (HREC no. 2014-062). Patients were evaluated preoperatively with complete clinical examination and imaging workup including a stereotactic MRI brain scan. Data consisted of *n* = 160 measurements, including 44 control sites, 55 arteries, and 61 veins. When restricted to vessels with a diameter of >500 μm and controls, there were *n* = 101 measurements, including 44 control sites, 14 arteries, and 43 veins.

### Handheld imaging needle

The needle comprises a standard outer metal sheath (Passive Biopsy Needle Kit, Medtronic, USA), encasing a custom-built imaging stylet. The stylet incorporates a side-viewing, fiber-optic probe connected to a spectral-domain OCT system (Telesto II, Thorlabs, USA). In clinical practice, the imaging stylet would be removed and replaced with the standard hollow stylet for biopsy tissue cutting once the needle was safely positioned at the point of biopsy. Use of the standard outer metal sheath (which includes fiducials for optical tracking) enabled easy calibration of the imaging probe with the stereotactic guidance system (StealthStation, Medtronic, USA) and allowed us to record the position of the probe during OCT acquisitions, relative to the preoperative MRI.

The imaging probe comprised a 3-m length of single-mode optical fiber (SMF-28-J9, Thorlabs, USA). At the distal end of the fiber, we fabricated focusing optics as described in (*41*). Briefly, a 350-μm length of no-core fiber (Success Prime Corporation, Taiwan) and 150 μm of graded-index (GRIN) fiber (DrakaElite, The Netherlands) were spliced to the single-mode fiber (SMF) to produce a weakly focused optical beam. This was terminated with a section of no-core fiber, angle polished at 52°, that redirected the light beam approximately perpendicular to the fiber using total internal reflection (TIR) at the fiber-air interface. The optical assembly was encased within a glass capillary and rigidly affixed with optical adhesive (NOA86H, Norland, USA) within a metal stylet (outer diameter, 2 mm; Minitubes, France) that had the same diameter as the standard Medtronic stylet (Passive Biopsy Needle Kit, Medtronic, USA). The glass capillary was necessary to maintain the TIR fiber-air interface once the probe was embedded in optical adhesive. The probe was positioned so that the light beam was emitted through a small imaging window (diameter, 500 μm), which had been drilled in the wall of the metal stylet. This was then oriented to perform imaging through the 5-mm (length) tissue-cutting window in the outer metal sheath of the biopsy needle.

Before use, the imaging probe was cleaned in a medical-grade washing machine (Steelco SpA, Italy), dried, and double bagged in Tyvek (DuPont, Virginia, USA), a high-density polyethylene. It was then sterilized using STERRAD (Advanced Sterilization Products, USA), a low-temperature, hydrogen peroxide gas plasma technique. Each probe was discarded after one surgery.

### OCT imaging

The imaging probe was interfaced proximally to a commercial spectral-domain OCT system (Telesto II, Thorlabs, USA) with a central wavelength of 1300 nm and an axial resolution of 5.5 μm (in air). During each neurosurgical procedure, OCT A-scan measurements were acquired at a rate of 23 kHz as the needle was manually moved over or inserted through the tissue.

### Automated vessel detection

In coherent imaging techniques, such as OCT, speckle arises because of the simultaneous backscatter of light from multiple subcellular components within a single imaging resolution element. The combined reflections may interfere constructively or destructively, giving rise to bright and dark speckles (*68*). As the tissue moves, such as movement of red blood cells in a vessel, the collection of subcellular components changes, and this alters the interfered OCT signal. Blood vessels, containing moving particles, appear as a region of rapid speckle decorrelation, and this has been used to identify vasculature (*54*, *69*–*71*). The rate of change in the OCT signal is related to the speed of movement, allowing differentiation of the rapid movement of red blood cells from the comparatively slow movement arising from insertion of the needle. Note that the degree of speckle decorrelation is a function of the quantity of moving red blood cells within the imaging light beam, not the vessel size. Thus, a dense collection of small vessels will give comparable speckle decorrelation to a single larger vessel with similar total flow.

Assessment of the rate of speckle decorrelation is complicated by a number of probe- and tissue-related factors, including the width of the imaging light beam, the signal-to-noise ratio of optical backscattering, and the presence of multiple scattering in the OCT signal. To correct for these confounding factors, we devised an algorithm that computed speckle decorrelation as a function of depth within the tissue and normalized against a control measurement taken over stationary, nonvascular brain tissue. A single control measurement was used for analysis of all patient scans.

We defined a 2D coordinate system, where *x* indexes the sequence of A-scans and *z* is oriented in the direction of the light beam (i.e., along the A-scan), such that the *x*-*z* plane defines an OCT B-scan. Given a subset of closely spaced A-scans as input data (a sequence of 500 A-scans was empirically chosen in this application), we calculated the square root of the normalized sum of squared intensity differences as a function of distance *dx* between the A-scans. This may be written as the square root of the expected value of the sum of squared intensity differences

$$f(dx)=\sqrt{E[{\left(I(x,z)-I(x+dx,z)\right)}^{2}]}$$ (1)

where *E*[] denotes the expected value of a function, *I*(*x*, *z*) is the OCT intensity at (*x*, *z*), *x* is taken over all A-scans in the subset, and *z* is taken over a small, fixed window size within which the OCT signal does not notably attenuate. For small *dx* values, *f*(*dx*) will be small with intensities highly correlated. The value of *f*(*dx*) will increase with increasing *dx* until it reaches a saturation value where there is negligible correlation between corresponding values in a pair of distant A-scans. We normalized these values to the range [0…1] by dividing them by the median value of *f*(*dx*) for *dx* greater than any anticipated correlation in the A-scan data. This gives *d*(*dx*), a normalized measure of speckle decorrelation between pairs of A-scans that are a distance *dx* apart

$$d(dx)=\frac{f(dx)}{\text{median}\{f(dx_{0}):n_{1}<dx_{0}<n_{2}\}}$$ (2)

where *n*_1_ and *n*_2_ are lower and upper bounds of a range of values of *dx* over which A-scans are highly decorrelated (100 and 400, respectively, in our application). Calculating *d*(*dx*) over a range of values for *dx* (1…40 in our application), we obtained a decorrelation curve that quantifies the increase in decorrelation between increasingly distant A-scans.

We acquired a control scan by rolling the imaging needle over a nonvascular region of exposed cortical tissue three times, providing a dataset of approximately 600,000 A-scans from which we calculated the decorrelation curve for control tissue. This dataset was used for evaluation of all other OCT scans in the study. As speckle decorrelation varies with imaging depth due to factors such as multiple scattering, we calculated a sequence of curves at increasing depths below the tissue surface. Analysis of OCT test data was performed against a curve of the appropriate depth taken from this control scan.

The rate at which the curve increases from 0 (highly correlated intensity values) to 1 (decorrelated intensity values) will increase in the presence of blood flow, as closely positioned A-scans will be less correlated due to movement of the red blood cells during acquisition of the A-scans. To detect the presence of a blood vessel in a new OCT dataset, the decorrelation curves were first calculated for each subset of data and at each depth. Each curve was then linearly scaled in the *x* direction to best fit the control curve (using the Euclidean *L*^2^-norm). Let us denote the scaling factor as *s*. The amount of scaling *s* quantifies the speckle decorrelation compared to stationary, control tissue. Stationary tissue required little scaling from the control scan (*s* ≈ 1), while areas of rapid blood flow generated a decorrelation curve that is scaled in proportion to the rate of blood flow (*s* >> 1). A blood vessel was detected when the average scaling factor (taken across all subsets in the dataset) was above an empirically selected threshold value.

The sensitivity and specificity values shown in Table 1 are for specific threshold values. A different threshold value was chosen when the analysis was restricted to large vessels (diameter, >500 μm) or all vessels. However, within each analysis, the same threshold value was used across all datasets and across all individuals. ROC curves were obtained by calculating sensitivity and specificity as the threshold value was varied. In the speckle decorrelation images shown in Figs. 3 to 8, we constructed an image where large values of *s* were graphically represented as bright pixels (white and yellow), while small values of *s* were represented by dark pixels (dark red).

For the three deep vessel insertions, the blood vessels were detected with an automated algorithm that used a simple pipeline of image processing techniques. The algorithm first calculated the mean speckle decorrelation (specified earlier by the scaling factor *s*) within each A-scan and smoothed these values between adjacent A-scans with a median filter. Smoothed flow values above a threshold value were marked as indicating vessels. Vessels smaller than a predefined number of A-scans were discarded. In all three acquisitions, the target vessel was successfully delineated.

### Preoperative MRI protocol for deep insertion acquisitions

For the three deep vessel insertion subjects, preoperative MRI scans with gadolinium contrast agent were acquired to identify target vessels and plan the insertion trajectory of the imaging needle. For subjects 1 and 3 (Figs. 6 and 8), scans were acquired with a Siemens 1.5T Aera using the following protocol: T1-weighted, gradient-eco sequence (MP RAGE), repetition time (TR) = 1910 ms, echo time (TE) = 3.14 ms, inversion time (TI) = 1100 ms, flip angle = 15°, slice thickness = 1 mm, and pixel size = 0.98 mm by 0.98 mm. For subject 2 (Fig. 7), scans were acquired on a Philips Healthcare 3T Ingenia using the following protocol: T1 stealth sequence, TR = 6.005 ms, TE = 2.68 ms, flip angle = 8°, slice thickness = 1 mm, and pixel size = 1 mm by 1 mm.

### Intraoperative optical data acquisition

During the superficial vessel protocol, craniotomy and durotomy were first performed, which exposed the network of vessels on the cerebral cortical surface. For each vessel measurement, a sterile scale bar was placed near the vessel and the imaging needle was rolled across the vessel to simulate needle movement during acquisition of the OCT signal. The needle was rolled rather than dragged across the vessel to reduce the risk of inadvertent hemorrhage. An intraoperative photograph was acquired showing the imaging needle, vessel, and scale bar to enable later analysis of vessel diameter. The vessel was also identified as either arterial or venous by the neurosurgeons (H.R. and C.R.P.L.). During OCT acquisition, our bespoke acquisition and analysis software displayed the OCT image to the neurosurgeon in real time, overlaid with measurements of the rate of speckle decorrelation. Areas of high speckle decorrelation were color coded (white and yellow) to provide an intraoperative indication of the presence of blood flow. These measurements were then repeated in negative control locations that showed no visible sign of vasculature.

An additional deep vessel protocol was performed on three patients before the superficial vessel protocol. All three patients had grade IV astrocytomas. A vessel adjacent to the tumor was identified on the preoperative MRI scan. A single burr hole was made, and the imaging needle was manually inserted to the depth of the vessel under stereotactic guidance (StealthStation, Medtronic, USA), acquiring OCT A-scans during the entire insertion. Once we had inserted to the planned depth, the imaging needle was rotated to confirm whether a blood vessel was adjacent to the needle. Rotation of the imaging needle was necessary, as earlier work has established that stereotactic guidance alone may lack sufficient accuracy to identify on which side of the needle the vessel will be located (*13*). The OCT scans, shown as insets in Figs. 6 to 8, were acquired once the needle had been rotated at a depth to image the vessel and then as the needle was gradually pulled back and removed. The OCT scans acquired during initial insertion were of comparable quality.

### Analysis of gold standard intraoperative photographs

A high-quality photograph (camera: Nikon D3, 12 megapixels, digital single-lens reflex) of each superficial vessel was acquired during OCT imaging, with a sterile scale bar positioned on the brain adjacent to the vessel. Vessel diameters were measured from the photographs using ImageJ (*49*), with the dimensions normalized against the sterile scale bar to give an absolute measurement in fractions of a millimeter. Spatial resolution of the photographs was approximately 42 μm per pixel.

### Statistical analysis

ROC curves were used to measure the accuracy of the OCT in discriminating vessels from controls. AUC, corresponding 95% CIs, and *P* values were generated to quantify the level of discrimination and the probability that the observed sample AUC is true. Threshold cutoffs were based on Youden’s index derived from sensitivity and specificity values of the ROC curves. Fisher’s exact two-sided tests were conducted between vessel type (all vessels and vessels >0.5 mm in diameter) as per gold standard photographic identification of vessels and speckle decorrelation against threshold values. A Mann-Whitney *U* test was run to determine flow difference between controls and vessels. Data were analyzed using IBM SPSS version 24.0 (Armonk, NY). *P* values <0.05 were considered statistically significant.

## Supplementary Material

http://advances.sciencemag.org/cgi/content/full/4/12/eaav4992/DC1

Download PDF

Intraoperative detection of blood vesels with an imaging needle during neurosurgery in humans

## SUPPLEMENTARY MATERIALS

Supplementary material for this article is available at http://advances.sciencemag.org/cgi/content/full/4/12/eaav4992/DC1 Fig. S1. MRI scans for deep vessel insertion #1 (corresponding to the OCT scan shown in Fig. 6). Fig. S2. MRI scans for deep vessel insertion #2 (corresponding to the OCT scan shown in Fig. 7). Fig. S3. MRI scans for deep vessel insertion #3 (corresponding to the OCT scan shown in Fig. 8).
