# Supplementary material for: Intraoperative detection of blood vessels with an imaging needle during neurosurgery in humans
Source: Sci Adv. 2018 Dec 19;4(12):eaav4992. doi: 10.1126/sciadv.aav4992 (PMC6300404; doi:10.1126/sciadv.aav4992)
Supplement: http://advances.sciencemag.org/cgi/content/full/4/12/eaav4992/DC1 [file supp_4_12_eaav4992__index.html]

Science Advances | Science AdvancesAAASSearchScience AdvancesMenu

## Supplementary Materials

**This PDF file includes:**

- Fig. S1. MRI scans for deep vessel insertion #1 (corresponding to the OCT scan shown in Fig. 6).
- Fig. S2. MRI scans for deep vessel insertion #2 (corresponding to the OCT scan shown in Fig. 7).
- Fig. S3. MRI scans for deep vessel insertion #3 (corresponding to the OCT scan shown in Fig. 8).

Download PDF

**Files in this Data Supplement:**

- Adobe PDF - aav4992\_SM.pdf
