# Supplementary material for: Intraoperative detection of blood vessels with an imaging needle during neurosurgery in humans
Source: Sci Adv. 2018 Dec 19;4(12):eaav4992. doi: 10.1126/sciadv.aav4992 (PMC6300404; doi:10.1126/sciadv.aav4992)
Supplement: Download PDF [file aav4992_SM.pdf]

Science Advances
AAAS

advances.sciencemag.org/cgi/content/full/4/12/eaav4992/DC1

Supplementary Materials for

Intraoperative detection of blood vessels with an imaging needle during neurosurgery in humans

Hari Ramakonar, Bryden C. Quirk, Rodney W. Kirk, Jiawen Li, Angela Jacques, Christopher R. P. Lind, Robert A. McLaughlin*

*Corresponding author. Email: robert.mclaughlin@adelaide.edu.au

Published 19 December 2018, *Sci. Adv.* **4**, eaav4992 (2018)
DOI: 10.1126/sciadv.aav4992

This PDF file includes:

Fig. S1. MRI scans for deep vessel insertion #1 (corresponding to the OCT scan shown in Fig. 6).
Fig. S2. MRI scans for deep vessel insertion #2 (corresponding to the OCT scan shown in Fig. 7).
Fig. S3. MRI scans for deep vessel insertion #3 (corresponding to the OCT scan shown in Fig. 8).

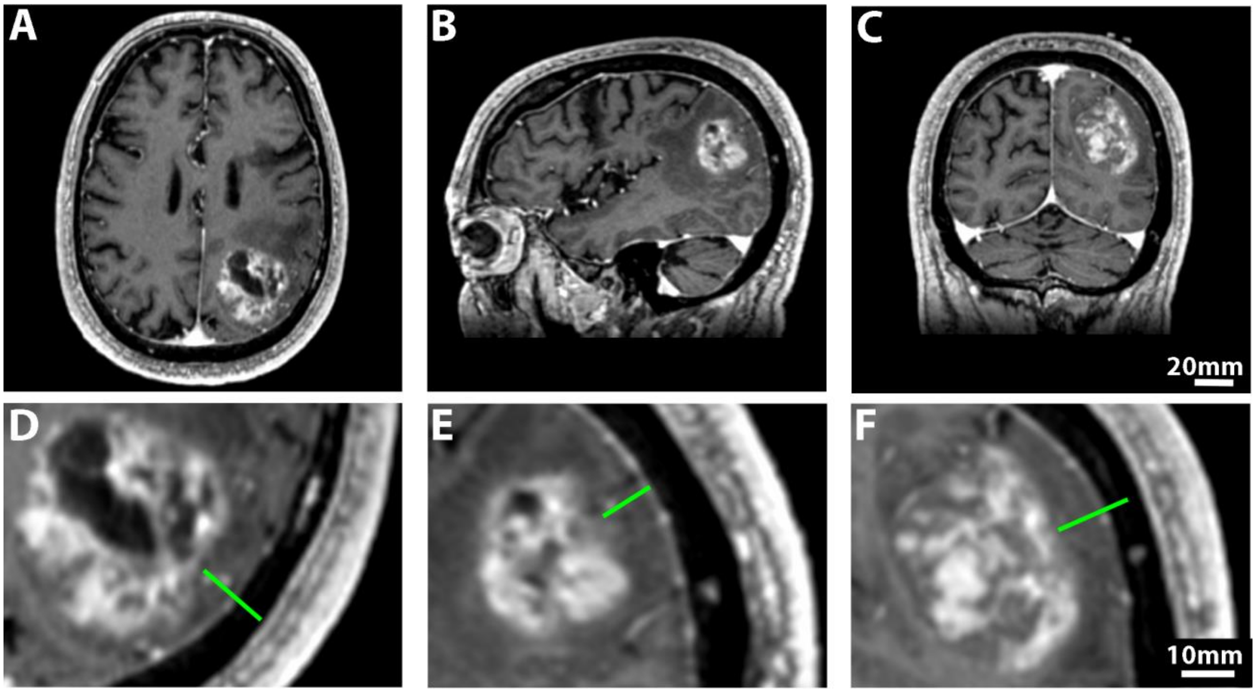

Fig. S1. MRI scans for deep vessel insertion #1 (corresponding to the OCT scan shown in Fig. 6). (A to C) axial, sagittal and coronal of subject 1, corresponding to the deep vessel insertion shown in Fig. 6. Slices have been chosen to include the endpoint of the insertion trajectory. (D to F) zoomed sections axial, sagittal and coronal scans (respectively). Green line shows the needle insertion trajectory. Note that the green line does not extend fully to the outer surface of the skull because the trajectory passes out of these slices.

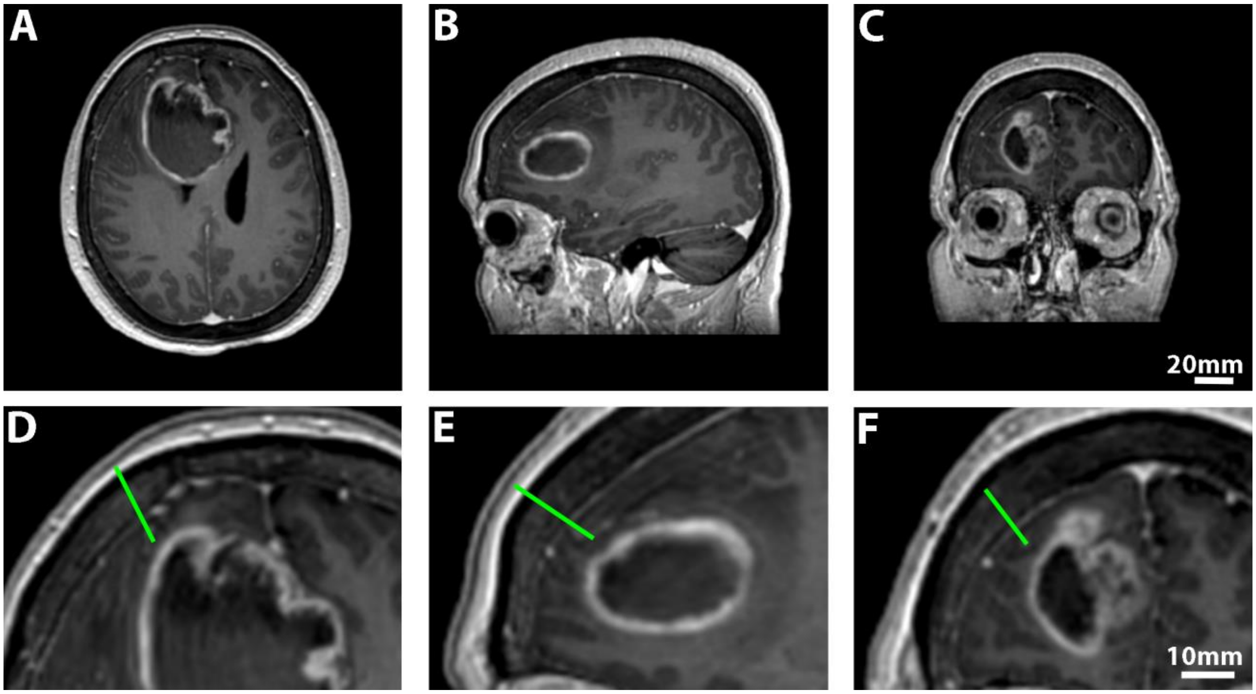

Fig. S2. MRI scans for deep vessel insertion #2 (corresponding to the OCT scan shown in Fig. 7). (A to C) axial, sagittal and coronal of subject 2, corresponding to the deep vessel insertion shown in Fig. 7. Slices have been chosen to include the endpoint of the insertion trajectory. (D to F) zoomed sections axial, sagittal and coronal scans (respectively). Green line shows the needle insertion trajectory. Note that the green line does not extend fully to the outer surface of the skull because the trajectory passes out of these slices.

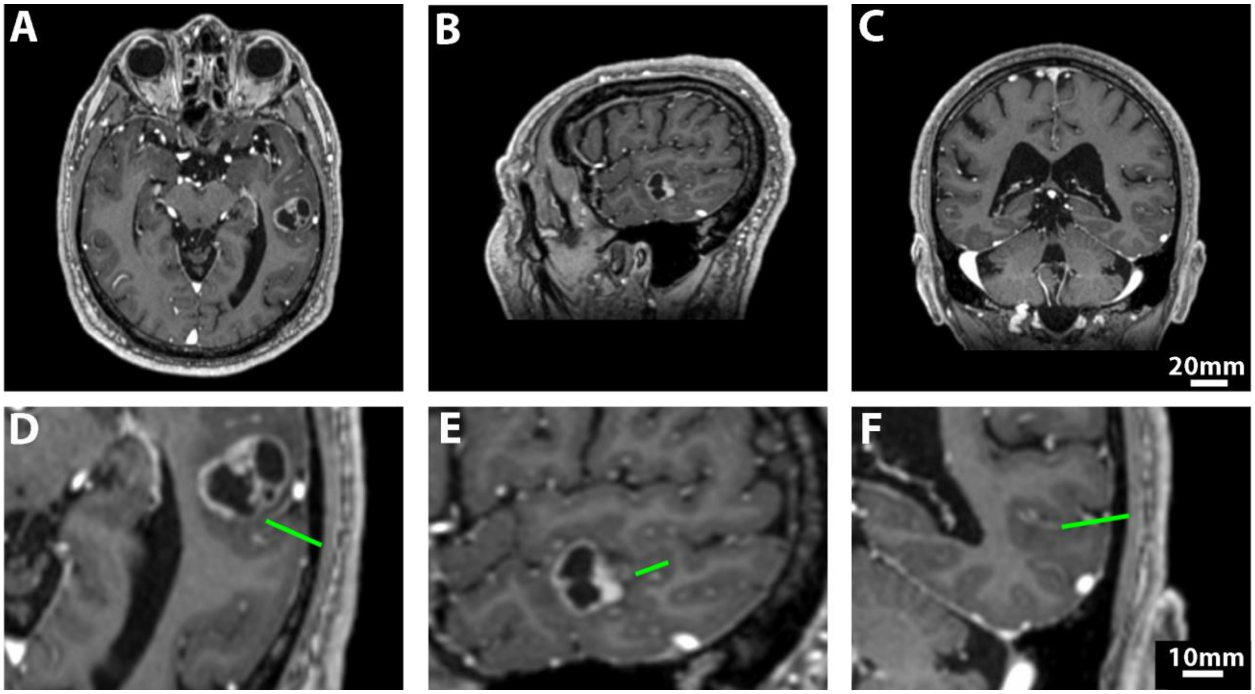

Fig. S3. MRI scans for deep vessel insertion #3 (corresponding to the OCT scan shown in Fig. 8). (A to C) axial, sagittal and coronal of subject 3, corresponding to the deep vessel insertion shown in Fig. 8. Slices have been chosen to include the endpoint of the insertion trajectory. (D to F) zoomed sections axial, sagittal and coronal scans (respectively). Green line shows the needle insertion trajectory. Note that the green line does not extend fully to the outer surface of the skull because the trajectory passes out of these slices. The vessel is posterior to the tumor, and so the tumor is not visible in the coronal slice.
